# Genetic analysis of first lactation production traits in Kankrej cattle

**DOI:** 10.14202/vetworld.2016.672-675

**Published:** 2016-06-29

**Authors:** K. J. Ankuya, N. K. Pareek, M. P. Patel, B. S. Rathod, K. B. Prajapati, J. B. Patel

**Affiliations:** Livestock Research Station, Sardarkrushinagar Dantiwada Agricultural University, Sardarkrushinagar, Gujarat, India

**Keywords:** correlation, heritability, first parity, Kankrej cattle

## Abstract

**Aim:**

The aim was to estimate genetic factors affecting the first lactation milk production traits in Kankrej cattle of North Gujarat.

**Materials and Methods:**

The 475 first lactation records of Kankrej cows that were maintained at the Livestock Research Station, Sardarkrushinagar Dantiwada Agricultural University, Sardarkrushinagar, Gujarat, over a period of 35 years from 1980 to 2014 were studied. The least squares maximum likelihood program was used to estimate genetic parameters of first lactation traits. Heritability was estimated through paternal half-sib analysis in adjusted data.

**Results:**

The heritability estimate for production traits was 0.40±0.17, 0.45±0.17, 0.35±0.18, and 0.20±0.14 for standard 300 days milk yield (F300Y), total lactation milk yield (FLY), wet average (FWA), and lactation length (FLL), respectively, in the first parity. All the genetic and phenotypic correlations among different production efficiency traits were high and positive. Genetic correlations between F300Y and FLY, FLL, and FWA were 0.80±0.20, 0.59±0.16, and 0.81±0.32, where as the phenotypic correlations were 0.969, 0.688, and 0.868, respectively. Genetic correlations of FLY with FLL and FWA were 0.60±0.13 and 0.79±0.20, whereas the phenotypic correlations were 0.777 and 0.817, respectively. Genetic and phenotypic correlation between FLL and FWA was 0.63±0.28 and 0.31, respectively.

**Conclusion:**

The heritability estimate of all first parity lactation traits waslow to medium (0.20-0.45) indicated the scope for further improvement in this trait through selection as well as managemental practice. Higher genetic and phenotypic correlation between thefirst lactation milk production traits gives theidea that genetic gain due to selection for one trait also givesmorecorrelated response of selection for other traits which is economically advantageous.

## Introduction

Kankrej cattle breed of India is considered as the best dual purpose cattle breed, i.e., female animals are used for milk production as well as male used for draught purpose in temperate region. Cattle in the tropics have, on average, lower milk yields and shorter lactations length than cattle in temperate countries. The difference is due to genetic and non-genetic factors.

Information of first lactation trait enables the breeder to predict the later lactation performance of the animals as it is highly correlated with the future performance traits [1].

The genetic composition of a population can be studied by considering the relative importance of heredity and environmental factors affecting the performance of individuals in that population [2]. Precise and accurate knowledge of genetic parameters is of paramount importance for planning appropriate selection and breeding strategies for the genetic improvement of dairy animals [3]. A basic prerequisite for planning of breeding program is the variability existing in the population and how much of this is caused by differences in the genetic make-up of the individuals. A quantitative measure of this is provided by heritability. With the help of heritability, one can predict the breeding value of the individual. The magnitude of heritability dictates the choice of the selection method and breeding system [4].

The present investigation was made to explore the genetic parameters such as heritability, genetic, and phenotypic correlations between first lactation milk yield (FLY) of first lactation traits in Kankrej cattle. It is envisaged that this information will be useful for the formulation of future breeding strategy for the genetic improvement of Kankrej cattle.

## Materials and Methods

### Ethical approval

The data are collected from Livestock Research Station from the history sheet so no need of ethical approval.

### Structure and management

The study was conducted at the Livestock Research Station, Sardarkrushinagar Dantiwada Agricultural University, Sardarkrushinagar, Gujarat (India), about 27 km from Palanpur city (23.81° 24.70’ N 71.10° 73.00’ E). The herd was established, in 1978, with Kankrej cattle, and the animals were kept in loose housing system. Calves were grouped according to age or live body weight while the adult cows were divided into lactating, advance pregnant, and dry group according to their reproductive physiological status. Feed requirement was determined according to milk yield, pregnancy status, and body weight.

### Collection of data

The data on 475 normal first lactation records of Kankrej cattle sired by 75 sires maintained at over a period of 35 years from 1980 to 2014 were included in the present study. Data of abnormal lactations such as abortion, mastitis and below 150 days milk yield, specific or non-specific diseases, reproductive disorder, and physical injury were excluded from the study.

### Statistical analysis

The data of milk production were analyzed for heritability, genetic, and phenotypic correlation coefficients by the method of paternal half-sib correlation using mixed model least-squares and maximum likelihood (LSMLMW) computer program developed by Harvey [5]. Heritability was estimated through paternal half-sib analysis in adjusted data as per the following model:

Y_ij_= μ +S_i_+ e_ij_

Where, μ = is the population mean, Y_ij_ is the observation on j^th^ trait of the daughter of the i^th^ sire, Si= the effect of i^th^ sire, and e_ij_ = the random error peculiar to individual within the group.

### Genetic and phenotypic correlations

Genetic correlation between two groups was calculated by the following formula:


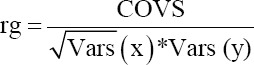


Where, rg is the genetic correlation, cov is covariance between sires, Vars (x) is the between sire variance of traits x, and Vars (y) is between sire variance of trait y.

The following formulae were used for calculating between and within sire variances:


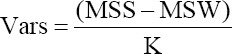


Varw = MSW

Where, MSS is the between sires mean squares, MSW is the within sire mean square, and K is the average number of progeny per sire.

Phenotypic correlation between two groups was calculated by the following formula:


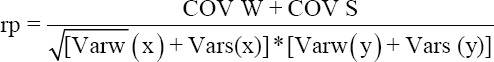


Where, rp is the phenotypic correlation, covw is phenotypic covariance within sires, covs is the phenotypic covariance between sires, Varw (x) is the phenotypic variance of traits x within sires, Vars (x) is phenotypic variance of trait x between sires, Varw (y) is phenotypic variance of trait y within sires, and Vars (y) is the phenotypic variance of trait y between sires.

The phenotypic variances between and within sire for different traits were calculated as mentioned for calculating heritability. Phenotypic covariance between sires and within sires was calculated using the following formulae:


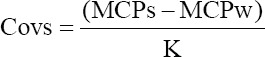


Covw = MCPw

Where, MCPw is mean cross products within sires and MCPs is mean cross products between sires.

## Results and Discussion

### Heritability, genetic, and phenotypic correlation between different milk production traits

The heritability estimates for first milk production traits are presented in Table-1, *viz*., first standard 300 days milk yield (F300Y), FLY, first wet average (FWA), and first lactation length (FLL)were 0.40±0.17, 0.45±0.17, 0.35±0.18, and 0.20±0.14, respectively.

**Table-1:** Genetic parameters for milk production traits in first parity of Kankrej cattle.

Traits	F300Y	FLY	WA	FLL
F300Y	0.40±0.17*	0.969	0.868	0.688
FLY	0.80±0.20	0.45±0.17*	0.817	0.777
WA	0.81±0.324	0.79±0.20	0.35±0.18*	0.311
FLL	0.59±0.16	0.60±0.13	0.63±0.28	0.20±0.14*

* Genetic correlation below diagonal, phenotypic correlation above diagonal. Heritability is bold. FLY=First lactation milk yield, F300Y=First standard 300 days milk yield, FLL=First lactation length

The heritability estimate for F300Y in the present study was 0.40±0.17, which indicates that 40% of variation in F300Y is due to heredity. Therefore, emphasis may be given in selection programs for improvement of this trait. The present estimate of heritability is comparable with the earlier findings in Tharparkar breed [6], Kankrej breed [7], and Holstein-Friesian cattle [8]. A lower estimate of heritability compared to present study was reported by other workers in Holstein-Friesian [9] and Brown Swiss cattle [10]. However, the heritability estimate in Sahiwal cattle was higher [11] and lower [12,13] that the present study.

### FLY

The heritability estimate of first lactation yield was medium (0.45±0.17) in Kankrej cattle, which is in accordance with the earlier findings in Tharparkar [6], Kankrej [7], and Jersey breed of cattle [14]. Lower heritability estimate compare to present study for FLY was reported in Sahiwal cattle [3,9,16], Holstein-Friesian [11], Brown Swiss cattle [12], and in Hariana cattle [15]. Medium heritability of FLY indicated that the selection on the basis of progeny performance would improve the first lactation yield and also point out that milk yield would increase by both genetic and managemental practice.

### FLL

The heritability estimate for FLL obtained in this study was low (0.20±0.14) in Kankrej cattle. The present estimate of heritability was comparable with earlier finding in the same breed [7] and in Sahiwal cattle [3]. Some of earlier workers reported lower estimate of heritability in Tharparkar cattle [6], Sahiwal cattle [9], and in Brown Swiss cattle [12]. The heritability of lactation length in dairy cattle is lower than many other economically important traits (FLY and F300Y). The low heritability of FLL illustrates that a major part of the variation in these characters are environmental, and selection would be not effective in bringing about genetic improvement, but improvement can be made through selection basis on the progeny performance and improving environmental conditions such as nourishing and management systems.

### FWA

Heritability estimate of FWA was 0.35±0.18 in present study. Choudhary *et al*. and Dhaka *et al*. reported lower estimate of heritability of FWA in Sahiwal breed [3] and Hariana breed [15] of cattle, respectively.

### Genetic and phenotypic correlation between milk production traits

#### F300Y and milk production trait

Genetic correlations between standard F300Y with total milk yield, FLL, and FWA were 0.805±0.206, 0.590±0.167, and 0.807±0.324, whereas the phenotypic correlations were 0.969, 0.688, and 0.868, respectively, which were all positive and highly significant indicating that higher F300Y were associated with higher FLY on phenotypic scale essentially due to both genetic and environmental reasons. Similar positive correlations were obtained in Tharparkar cattle [6], in Kankrej breed [7], in Sahiwal cattle [9], and in HF cattle [11]. High genetic correlations amongF300Y and the other efficiency traits are obvious because the latter is derived from F300Y, i.e. all the efficiencytraits have F300Y as numerator. High genetic correlations among these traits indicated simultaneous improvement in other traits while selecting anyone of them. Very high correlations of F300Y with FLY showed that sires can be evaluated for milk yield on either of the trait and improvement on one trait could bring a concomitant increase on the other. The positive and significant correlations between F300Y and FLY indicated that higher FLYs on total lactation length as well as on 300 days lactation basis were associated with longer lactation period in Kankrej cows and selection for higher F300Ywill increase total lactation yield as well as the length of lactation period in first parity.

#### FLY and milk production trait

FLY had high positive genetic and phenotypic correlation with FLL and FWA. The genetic and phenotypic correlations between FLY and FLL were 0.60±0.13and 0.777 and with FWA were 0.79±0.20 and 0.817, respectively. The present estimate of the correlation between the FLY and FLL is comparable to the estimates reported by earlier in Kankrej cattle [7], Brown Swiss cattle [12], Sahiwal cattle [16-18], and in Holstein-Friesian cattle [19] and with FWA comparable in Hariana cattle [15]. The genetic correlation of 0.60 indicated the plieotropic effect of the genes on the two traits. It also suggested that lactation length and the wet average would be increased as a correlated response to selection for milk production.

#### FLL and FWA

The genetic and phenotypic correlations between FLL and FWA were 0.63±0.28 and 0.311, respectively.

## Conclusion

FLY was positively correlated with the other production traits, which indicated that any increase in slandered 300 days milk yield or lactation length would simultaneously bring about an increase in total lactation milk yield which could be due to the same set of genes responsible for the expression of these traits. A positive and significant genetic correlation between standard lactation milk yield and other milk production traits under study giveidea that selection for higher Standard lactation milk yield will increase the lactation length and wet average which is economically advantageous. The medium heritability estimate of FLY and FWA indicated the wide scope for further improvement in this trait through selection. However, lactation length likely to achieve slow progress due to its low heritability. As a result of the moderate heritability estimates for total lactation milk yield and standard 300 days milk yield, it can be concluded that genetic development of milk yield can be attained through selective breeding. Therefore, these two traits may be preferred by breeders as selection criteria for development of effective genetic improvement program.

## Authors’ Contributions

The data on first lactation production traits were collected by KJA, MPP, and BSR under the supervision of KBP and JBP. The data were compiled and analyzed by NKP. The manuscript was prepared by NKP and MPP under guidance of KBP. All the authors read and approved the final manuscript.
